# Age-related episodic memory decline and the role of amyloid-β: a systematic review

**DOI:** 10.1590/1980-57642021dn15-030002

**Published:** 2021

**Authors:** Jandirlly Julianna Souto, Gabriella Medeiros Silva, Natalia Leandro Almeida, Irina Ivanovna Shoshina, Natanael Antonio Santos, Thiago Paiva Fernandes

**Affiliations:** 1 Department of Psychology, Universidade Federal da Paraíba – João Pessoa, PB, Brazil.; 2 Perception, Neuroscience and Behaviour Laboratory, Universidade Federal da Paraíba – João Pessoa, Brazil.; 3 Laboratory of Physiology of Vision, Pavlov Institute of Physiology – St. Petersburg, Russia.

**Keywords:** memory, episodic, aging, healthy aging, beta amyloid, systematic review, memória, memória episódica, envelhecimento, envelhecimento saudável, peptídeos beta-amiloides, revisão sistemática

## Abstract

Aging has been associated with the functional decline of episodic memory (EM). Unanswered questions are whether the decline of EM occurs even during healthy aging and whether this decline is related to amyloid-β (Aβ) deposition in the hippocampus.

**Objective:**

The main purpose of this study was to investigate data on the relationship between the age-related EM decline and Aβ deposition.

**Methods:**

We searched the Cochrane, MEDLINE, Scopus, and Web of Science databases and reference lists of retrieved articles that were published in the past 10 years. The initial literature search identified 517 studies. After screening the title, abstract, key words, and reference lists, 56 studies met the inclusion criteria.

**Results:**

The overall results revealed that increases in Aβ are related to lower hippocampal volume and worse performance on EM tests. The results of this systematic review revealed that high levels of Aβ may be related to EM deficits and the progression to Alzheimer’s disease.

**Conclusions:**

We discussed the strengths and pitfalls of various tests and techniques used for investigating EM and Aβ deposition, methodological issues, and potential directions for future research.

## RESUMO

O envelhecimento tem sido associado a um declínio funcional da memória episódica (ME). Algo ainda sem resposta é se o declínio da ME ocorre mesmo no envelhecimento saudável e se esse declínio pode estar relacionado à deposição de Aβ no hipocampo.

**Objetivo:**

Nosso objetivo principal foi investigar os dados sobre a relação entre a memória episódica e a deposição de Aβ no envelhecimento saudável.

**Métodos:**

Nós buscamos nas bases de dados Cochrane, MEDLINE, Scopus, Web of Science e nas listas de referências dos estudos dos últimos 10 anos. A busca inicial nas bases de dados identificou 517 estudos. Após a triagem de título, resumos, palavras-chave e referências, 56 estudos atenderam aos critérios de inclusão.

**Resultados:**

O resultado geral revelou que o aumento de Aβ estava relacionado ao menor volume do hipocampo e pior desempenho em testes de ME. Em resumo, os resultados da presente revisão sistemática revelaram que altos níveis de Aβ podem estar relacionados ao declínio de ME e conversão progressiva para a Doença de Alzheimer.

**Conclusões:**

Aqui, discutimos os pontos fortes e as limitações dos testes e técnicas para investigar a deposição ME e Aβ, bem como questões metodológicas e direções futuras.

## INTRODUCTION

Aging has been associated with a functional decline in episodic memory (EM). An assessment of the memory function in healthy aging can represent a meaningful alternative since Alzheimer’s disease (AD) tends to be diagnosed at more advanced stages, especially when memory impairments appear. Approximately, 30% of elderly individuals with normal aging, above 60 years old, can have high levels of amyloid-β (Aβ) deposition in the hippocampus.^1^ Hence, they tend to be more likely to develop AD.^2^

Age-related memory decline, especially in those related to EM, is common in aging, and Aβ deposition has been associated with a progressive conversion to AD.^3^ EM can be understood as the integration of the “what,” “when,” and “where” (or “which”) components, which is a substantial factor in the social life of an individual.^4^ However, it is unclear whether hippocampal volume atrophy and age-related EM decline in healthy aging are entirely related to the deposition of Aβ.^5^ One of the reasons is because there are links between the neocortex and the hippocampus. In this way, it is essential to understand the relationship of the hippocampus with Aβ deposition to further unravel the other areas related to EM.^3^

The hypothetical model of biomarkers in AD indicated that the presence of Aβ is related to the functioning of the vascular systems and growth.^6,7^ This model describes the integration of AD biomarkers, which may reflect an underlying pathophysiological sequence of the following events: (1) the presence of amyloid-β42 in the cerebrospinal fluid (CSF) is first detected (the same is true for tau blood biomarkers);^8^ (2) levels of tau protein in CSF are significantly increased; (3) hypometabolism of fluorodeoxyglucose occurs; (4) brain atrophy occurs; and (5) cognitive decline is noted.

The model of biomarkers in AD suggests that the relationship between Aβ and cognitive impairment is not immediately successive, and therefore it should be less evident than the relationship between neurodegenerative biomarkers and cognitive impairment.^8^ Notwithstanding, the deposition of Aβ before the clinical diagnosis of AD remains a challenge, since less, if any, relationship between a decline in cognition in healthy aging and the deposition of Aβ in the hippocampus can be observed. In this manner, it is comprehensible that cortical thickness exerts a substantial influence on Aβ deposition and the consequent decline in EM.^9^

Age-related dementia typically begins slowly and gradually with brain atrophy before the onset of clinical symptoms.^7,8,10^ Some authors have suggested that the cognitive decline in aging is related to undetected diseases and, hence, is not a characteristic of normal aging.^11^ An important implication of detectable brain atrophy and dementia is that some cases with undetected diseases can help create inferences about normal brain aging.^12^ This is of particular concern, since the proportion of elderly people with undetected neurodegenerative disease is expected to increase, potentially leading to baseless conclusions of accelerated age-related decline in the cortical areas vulnerable to disease pathology, especially in the entorhinal cortex and the hippocampus.^13^

Therefore, the contribution of latent pathology to age-related decline in healthy aging remains an open question. However, differences in AD biomarkers, such as brain atrophy and Aβ deposition, may clarify this issue. This review is based on the assumption that the presence of Aβ in people with healthy aging can serve as a prodromal state of AD. Thus, brain atrophy and Aβ deposition are biomarkers of the disorder. Here, our main purpose was to investigate the data on the relationship between EM and Aβ deposition in healthy aging.

## METHODS

### Search strategy

The PRISMA guidelines were used.^14,15^ Exhaustive electronic searches were conducted on the studies that were published from 2010 to 2021 in the following databases: COCHRANE, Medline, Scopus, and Web of Science (PROSPERO: CRD42020190981). The following search strategy was used: Aging OR Senescence OR Aged OR Elderly OR “Healthy Aging” OR “Aging Well” OR “Healthy Ageing” AND “Amyloid beta-Peptides” OR “Amyloid beta Peptides” OR “Alzheimer beta-Protein” OR “Alzheimer’s ABP” OR “beta-Amyloid Protein” OR “Amyloid beta Protein” OR “Amyloid Protein A4” OR “beta Amyloid” OR “Amyloid AD-AP” OR “Amyloid” OR “Amyloid Substance” OR “Amyloid Fibril” OR β-amyloid OR amyloid-β OR “amyloid β-peptide” OR “amyloid-beta” AND “Histology” OR “Histocytochemistry” OR “Immunohistochemistry” OR “Immunolabeling Technique” OR “Immunolabeling Technic” OR “Immunogold Technique” OR “Immunohistocytochemistry” OR Positron-Emission Tomography” OR “Positron Emission Tomography” OR “Positron Emission Tomography Imaging” OR “PET Scan” OR “PET Imaging” AND “Episodic Memory” OR “Autobiographical Memory” OR “Prospective Memory.” The key words were chosen even in the absence of a specific term (according to the MeSH) to prioritize sensitivity over the specified theme. In addition, we examined the reference lists in the retrieved studies.

### Selection criteria

We included studies that investigated Aβ deposition in healthy aging and its relationship with decline in EM.^16,17,18,19,20^ We adopted the following inclusion criteria: (a) we investigated Aβ in the hippocampus in individuals with healthy aging and (b) we used the tasks to assess EM. We excluded studies that (a) used animal models, (b) did not assess the hippocampus, (c) evaluated another type of memory instead of episodic, and (d) were literature reviews.

### Data extraction

For each study, data were extracted independently by two authors (GS and JS) using a structured form. The discrepancies were resolved by consulting a third author (NA) if needed. If there was insufficient information in the studies, the respective author was contacted. The following variables were extracted: (1) demographic and clinical characteristics (e.g., number of patients); (2) study design; (3) characteristics of the techniques; (4) task for assessing EM; and (5) main findings.

### Quality assessment

We performed individual and comprehensive quality assessments for each study. The studies were also evaluated based on internal validity (i.e., selection bias or attrition bias) and construct validity (i.e., adequacy of the operational criteria used). In general, the quality and evidence of the studies were assessed based on three main measures, namely, (a) limitations (e.g., poorly designed strategies), (b) consistency of the results, and (c) accuracy (i.e., ability to generalize findings and provide sufficient data). A quality assessment was conducted using the PEDro scale and the Appraisal Tool for Cross-Sectional Studies (AXIS).

## RESULTS

**Figure 1.**
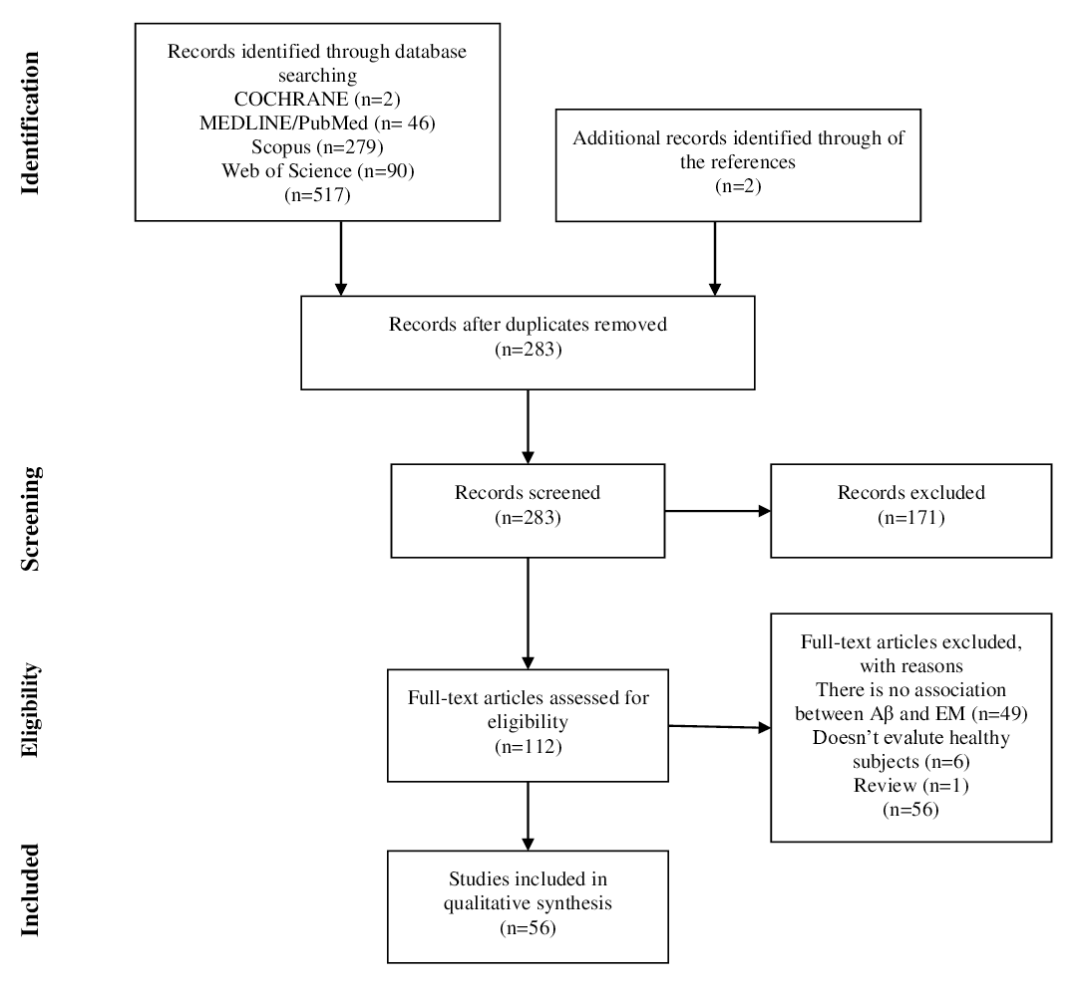
Flowchart of the present study.

The initial search of the databases identified 517 studies. After screening the title, abstracts, key words, and article references, a total of 56 studies were in compliance with the inclusion criteria. Figure 1 presents the diagram flow and the details used to identify studies in our review.

### General characteristics of the studies

Table 1 presents the selected studies, which were published between 2010 and 2021. Thirty studies (53.6%) were published between 2010 and 2016, and the other 26 studies (46.4%) were published between 2017 and 2021. Regarding the sample size, the studies showed a variance between 45 and 2,908 participants, the majority (76.8%) being more than 100 participants.

**Table 1.** Characteristics of the selected studies.

Authors	Sample	Aβ technique	Others techniques	Regions of interest	EM task	Main findings	PEDro	AXIS
Chételat et al.^58^	93	PiB-PET	N/A	Gray matter, white matter, and CSF	CVLT-II And RCFT	EM involvement is related to Aβ deposition, especially in the temporal neocortex, and regardless of hippocampal atrophy	4	15
Lim et al.^59^	141	PiB-PET	Blood exam	Cerebellum	CVLT-II and PAL	High Aβ showed significantly greater decline in verbal and visual EM at 18 months	5	15
Marchant et al.^60^	54	PiB-PET	MRI and FLAIR	CSF, WMH, frontal, parietal, and temporal cortex, posterior cingulate, and precuneus	CVLT and MAS	Aβ did not explain changes in EM	5	16
Perrotin et al.^61^	48	PiB-PET	MRI	Medial PFC/ACC, precuneus/PCC/ICC, and temporal lobe	CVLT-II and WMS-R	High Aβ performed worse than low Aβ just on the EM measure test CVLT	5	15
Rodrigueet al.^62^	137	F-florbetapir PET	Blood exam	–	HVLT and CANTAB	Aβ subgroup has no significant association with EM	5	16
Adamczuk et al.^63^	64	F-flutemetamol PET	MRI	Frontal, parietal, anterior cingulate, precuneus/posterior cingulate, and lateral temporal	BNT, AVF, and RPM	Aβ and EM were negatively correlated only in the BDNF group of met +ve/APOE ε 4 +ve	5	14
Doré et al.^64^	133	PiB-PET	MRI	Gray matter, white matter, CSF, and cerebellum	CVLT-II and LMT-II	No significant differences in EM between the Aβ- and Aβ+ groups. There was a significant reduction in the precuneus and hippocampus	5	13
Ellis et al. ^65^	178	PiB-PET	Blood exam	Frontal, superior parietal, lateral temporal, lateral occipital, and anterior and posterior cingulate	CVLT-II, LMR, and RCFT	High Aβ showed greater decline in EM	5	14
Hedden et al.^66^	168	PiB-PET	MRI and FLAIR	Frontal, lateral, parietal, temporal, and retrosplenial cortices	FNAME, STSRT, and MCT	Aβ burden and WMH had distinct cognitive profiles. Aβ was associated with a decline in EM	5	14
Lim et al.^67^	234	PiB-PET	Blood exam	Cerebellar cortex, frontal, superior parietal, lateral temporal, lateral occipital, and anterior and posterior cingulate	CVLT-II and RCFT	No differences were observed between HC high and low Aβ groups	4	15
Sperling et al.^68^	78	Florbetapir F 18 PET	MRI	Grey matter, frontal, temporal, parietal cortices, anterior cingulate, and posterior cingulate	WLM-I, WLM-D, and DSS	The highest SUVr correlated with the lowest immediate memory	8	16
Wirth et al.^69^	38	PiB-PET	FDG-PET and MRI	Frontal, temporal, parietal, and anterior/posterior cingulate	LMR and VRT	PiB positivity was associated with nonmemory decline	3	14
Lim et al.^70^	225	PiB-PET	Blood exam	Cerebellar cortex	CVLT-II	High Aβ HC and MCI groups showed moderate decline in EM	7	16
Lim et al.^71^	413	PiB-PET	Blood exam	Cerebellar cortex	LMR, CVLT-II, RCFT, and COCLT	Aβ+ showed greater decline on the verbal EM and visual EM	7	16
Ossenkoppele et al. ^72^	81	PiB-PET	FDG-PET and MRI	Gray matter, posterior cingulate cortex, bilateral angular gyri, and bilateral inferior temporal gyri	CVLT and VRT	Aβ was associated with higher metabolic activity and lower visual episodic memory scores	5	15
Villeneuve et al.^9^	67	PiB-PET	MRI	Right frontal, parietal, temporal, occipital, and precuneus	MAS	High Aβ was associated with cortical thinning and lower performance of EM	5	16
Amariglio et al.^73^	257	PiB-PET	FDG-PET and MRI	Hippocampal volume	FNAME, STSRT, and MCT	Aβ and ND biomarkers predict biggest changes in EM	8	16
Aschenbrenner et al.^74^	238	PiB-PET	Cerebrospinal fluid assessment	Orbitofrontal, parietal, frontal, frontal, and temporal	SRT	EM was not correlated with levels of Aβ	5	16
Lim et al. ^75^	227	PiB-PET	MRI	Gray matter, white matter, cerebrospinal fluid, and hippocampus	OCL	Aβ+ CN and MCI groups showed decline on the EM measures	6	15
Lim et al. ^76^	333	PiB-PET	Blood exam	Cerebellar cortex	LMR, CVLT-II, RCFT, and COCLT	Aβ+ ɛ4+ individuals showed cognitive decline across all domains. Aβ+ ɛ4− individuals showed faster decline only in verbal EM	8	17
Mander et al.^77^	26	PiB-PET	fMRI and EEG	Hippocampus and mPFC	TWPT	Aβ impairs sleep and has indirect implications for EM decline	8	18
Mattsson et al.^52^	743	Florbetapir PET	FDG-PET and MRI	Gray matter	LMR and AVLT	Aβ+ was associated with lower EM scores	4	16
Pietrzak et al.^78^	333	PiB-PET	–	–	CVLT and LMR	High Aβ was associated with a subtle decrease in EM	8	16
Racine et al.^79^	175	Electrochemiluminescence	MRI and FLAIR		RAVLT and WMS-RLM	High levels of Aβ and higher rates of decline in EM tests	4	18
Wang et al.^80^	188	CSF collection	MRI	Entorhinal cortex, fusiform gyrus, inferior, middle, and superior temporal gyri, superior and inferior parietal lobules, posterior cingulate gyrus, and precuneus	N/A	Reduced CSF Aβ42 was related to poorer performance on EM	3	16
Wang et al.^81^	263	PiB-PET	Blood exam	–	LMIRD and WLIRD	For asymptomatic carriers, Aβ burden was predictive of longitudinal decline in EM	8	15
Bischof et al.^82^	147	Florbetapir PET	–	–	HVLT and CANTAB	Higher Aβ may have an influence on EM, especially between 30 and 55 years of age	7	16
Lim et al.^83^	423	PiB-PET	Blood exam	Cerebellar cortex	CVLT, LMR, and RCFT	Significantly increased decline in EM in Aβ+ APOE ε4 carriers	7	17
Mielke et al.^84^	465	PiB-PET	–	–	AVLT, WMS-RLM, WMS-RVR, and CogState	High Aβ was not associated with changes in EM	5	16
Song et al.^85^	82	F18-AV-45-florbetapir PET	MRI and fMRI	–	HVLT and CANTAB	Aβ had no effects or interaction in EM	4	14
Ayton et al.^86^	117	PiB-PET		Hippocampus	CVLT and RCFT	Aβ pathology and higher levels of quantitative susceptibility of the hippocampus predicted accelerated deterioration in EM	6	17
Boots et al.^87^	140	PiB-PET	–	–	RAVLT	BDNF was associated with a decline in EM and was exacerbated by a greater Aβ load	6	17
Farrell et al.^88^	174	F-florbetapir PET	–	–	–	Aβ+ and the increase in baseline SUVR predicted an increasing decline in EM	7	16
Lim et al.^89^	989	PiB-PET	Blood exam and MRI	Hippocampus volume	CVLT-II, LMR, and RCFT	APOEɛ4 homozygotes (ɛ4/ɛ4) showed significantly worse EM and higher Aβ levels than heterozygous ɛ4	5	14
Lim et al.^90^	446	PiB-PET	Blood exam	Hippocampus volume	LMR, CVLT-II, and RCFT	Aβ- Val and Aβ+ Val homozygotes showed a decline in EM	4	17
Pietrzak et al.^91^	416	PiB-PET	MRI	Plasmatic cortisol	CVLT-II, LMR, and RCFT	Older Aβ+ adults experienced a faster decline in EM	3	16
Vogel et al.^92^	136	PiB-PET	–	–	N/A	Decline in EM was observed only when cognitive decline and Aβ were present	7	17
van Bergen et al.^93^	116	Flutemetamol PET	MRI	–	MMSE and VLMT	Local correlation between iron and β-amyloid is related to levels of cognitive performance	5	18
Bilgel et al.^94^	171	PiB-PET	MRI	Hippocampus	CVLT and BVRT	Amyloidosis or hippocampal atrophy had longitudinal declines in EM verbal and learning	3	17
Farrell et al.^95^	126	Florbetapir PET	MRI	Hippocampal and cortical volume	HVLIR and CANTAB	Decline in EM and increase in Aβ accumulation	7	18
Jansen et al.^96^	2,908	PiB-PET	–	–	VWLT	Aβ positivity was associated with low memory	6	17
Leal et al.^97^	71	PiB-PET	–	–	WMS - II and CVLT	Higher levels of Aβ are associated with the decline of EM	5	16
Lim et al.^98^	447	PiB-PET	MRI	–	RAVLT	Worsens in EM of Aβ+ than Aβ− and may exacerbate with age	5	18
Mecca et al.^99^	45	PiB-PET	MRI	Gray matter	N/A	In middle-aged individuals, Aβ load does not affect EM performance	6	18
Ko et al.^100^	762	PiB-PET	–	–	RAVLT and ADAS-Cog	EM was a predictor for Aβ positivity	6	17
Pothier et al.^101^	65	F-florbetapir PET	–	–	FCSRT	Significant difference for EM over time, with better performance in Aβ- compared with Aβ+.	8	19
Rabin et al.^102^	253	PiB-PET	MRI	Fornix	WMS-RLM, FCSRT, and STSRT	Elevated Aβ load has been associated with a faster decline in EM over time	4	17
Rahayel et al.^103^	104	PiB-PET	MRI	–	WMS - III and RAVLT	Higher Aβ had worse episodic memory	5	17
Yu et al.^104^	148	SRM	–	–	–	Aβ is unrelated with decline in EM	8	19
Dupont et al.^105^	104	PiB-PET	MRI	WMHs	LMT -II	Deposition Aβ predicts weaker EM performance	8	18
Joannette et al.^106^	104	PiB-PET	MRI	–	RAVLT	EM performance is associated with Aβ load	6	18
Lim et al.^107^	213	PiB-PET	Blood exam	–	CVLT and RAVLT	Aβ is related to the decline of EM	7	16
Lindbergh et al.^23^	149	Florbetapir PET		Gray matter	RAVLT and RCFT	Women with high Aβ are more vulnerable to declining EM than men	6	18
Squarzoni et al.^108^	108	PiB-PET			RAVLT	Aβ load and poorer memory performance were detected only in stages +	6	16
Busatto Filho et al.^109^	124	PiB-PET	MRI and FDG-PET	Hippocampal subregions	AVLT and SCPT	Subicular volumes were inversely correlated with the degree of Aβ deposition. Verbal EM scores were significantly lower in both (N)+ groups	5	15
Han et al.^110^	154	PiB-PET	MRI	VL and WS	SENAS	Aβ influence retention in EM change	6	19

6-TSRT: 6-Trial Selective Reminding Test; Aβ: amyloid-β; AD: Alzheimer’s disease; ADAS-Cog: Alzheimer’s Disease Assessment Scale–Cognitive Subscale; APOE: apolipoprotein E; AVF: Animal Verbal Fluency Test; AVLT: Auditory Verbal Learning Test; BDNF: brain-derived neurotrophic factor; BNT: Boston Naming Test; BVRT: Benton Visual Retention Test; CAA: cerebral amyloid angiopathy; CANTAB: Cambridge Neuropsychological Test Automated Battery; CSF: cerebrospinal fluid; COBT: Cogstate One Back Task; COCLT: Cogstate One Card Learning task; CVLT-II: California Verbal Learning Test, second edition; DMST: Delayed Matching-to-Sample Task; DTI: diffusion tensor imaging; EEG: electroencephalogram; ELISA: enzyme-linked immunosorbent assay; EM: episodic memory; FCSRT: Free and Cued Selective Reminding Test; FDG: fluorodeoxyglucose; FLAIR: fluid attenuation inversion recovery; fMRI: functional magnetic resonance imaging; FNAME: Face–Name Associative Memory Exam; FNET: Face–Name Experimental Task; FSRT: Free and Selective Reminding Test; HVLIR: Hopkins Verbal Learning Immediate Recall; HVLT: Hopkins Verbal Leaning Task; PiB-PET: Pittsburgh compound B positron emission tomography; IA: immunosorbent assay; LMT-II: Logical Memory Test, second edition; LMR: Logical Memory Recall; LMIR: Logical Memory Immediate Recall and Delayed Recall; MAC-Q: Memory Complaint Questionnaire; MAS: Memory Assessment Scale; MBT: Memory Binding Test; MMSE: Mini Mental State Examination; MPRAGE: magnetization-prepared rapid gradient echo; MRI: magnetic resonance imaging; MCT: Memory Capacity Test; mPFC: medial prefrontal cortex; MR: magnetic resonance; MTL: medial temporal lobe; N/A: no data available; OCL: One Card Learning; PAL: paired-associate learning; P-tau: tau pathology; RAVLT: Rey Auditory Verbal Learning Test; RCFT: Rey Complex Figure Test; ROCF: Rey-Osterrieth complex figure; RPM: Raven’s Progressive Matrices; SCC: subjective cognitive concerns; SCPT: Short Cognitive Performance Test; SENAS: Spanish and English Neuropsychological Assessment Scale; SMD: subjective memory decline; SRM: selected reaction monitoring; SRT: Selective Reminding Test; STSRT: Six-Trial Selective Reminding Test; SUVR: neocortical standardized uptake value ratio; TMADA: TaqMan allelic discrimination assay; TWPT: the word-pairs task; VPATS: Visual Paired Associates Total Score; VL: vascular load; VRM: verbal recognition memory; VRT: Visual Reproduction test; VWLT: Verbal Word Learning Test; WAIS: Wechsler Adult Intelligence Scale; WLM-V: Wechsler Logical Memory–Delayed Recall; WM: working memory; WMS-RLM: Wechsler Memory Scale-Revised Logical Memory; WMS-RVR II: Wechsler Memory Scale-Revised Visual Reproductions II; WLIRD: Word List Immediate Recall and Delayed Recall; WLM-I: Wechsler Logical Memory I and II Story A Immediate; WMH: white matter hyperintensities.

A longitudinal study design was used in almost all of the selected studies (71.4%), whereas eight studies (28.6%) employed a cross-sectional design. Follow-up in these studies ranged between 6 months and 23 years. Diagnostic criteria also varied between studies, that is, 24 studies used the Mini-Mental State Examination (85.7%), 16 studies used the Clinical Dementia Rating (57.1%), 12 studies used the Geriatric Depression Scale (42.8%), and 5 studies used the Hospital Anxiety and Depression Scale (17.8%).

### Techniques for investigating Aβ

We observed four types of techniques for investigating Aβ. The main technique used was positron emission tomography (PET), performed in 53 studies (94.6%). Three types of radioligands were used for the PET technique. Pittsburgh compound B was used in most of the studies (75%), while florbetapir F 18 and flutemetamol F 18 were used by a limited number of studies, i.e., nine (16%) and two (3.6%) studies, respectively. In addition, electrochemiluminescence techniques (in one study, 1.8%), CSF collection (in one study, 1.8%), and selected reaction monitoring (in one study, 1.8%) were also used.

### Tasks used for the assessment of episodic memory

Most studies (67.9%) utilized between two and four different neuropsychological tests to assess EM. Approximately, 25 of these studies (44.6%) concentrated their assessments based on two types of tests. Predominantly, most of them used neuropsychological measures, such as California Verbal Learning Test (CVLT; 35.7%), Rey Complex Figure Test (RCFT; 19.6%), Logical Memory Recall (17.8%), Rey Auditory Verbal Learning Test (16%), and the Wechsler Memory Scale-Revised (12.5%). Five studies did not specify the tests used in the neuropsychological assessment.

### Main findings

The main findings are presented in Table 1. The scope of the selected studies included heterogeneous objectives.^21,22^ Nevertheless, all the studies evaluated common aspects of Aβ deposition in cortical structures, especially in the hippocampus, and its relationship with EM. Hence, the results have specificities. In general, most studies (83.9%) indicated that the increase in Aβ was related to worse performance in EM tests. One of these studies even indicated that Aβ deposition in women makes them more vulnerable to declines in EM.^23^ In contrast, some studies (16.1%) indicated that the increase in Aβ did not correspond to differences in EM.

### Quality assessment

Based on the PEDro scale, which comprised 11 items, 56 studies obtained an overall average of 5.7 points, approaching the score considered by the moderate- to high-quality instrument (≥6.0 points — moderate to high quality). Overall, 53.6% were considered of moderate quality (4.0–5.0 points) and the other 46.4% were of moderate to high quality (6.0–9.0 points). Based on the AXIS, the overall average was 16.2 points on a scale ranging from 0 to 20 points. Overall, 28.6% (16 studies) scored from 0 to 15 points and the other 71.4% (40 studies) reached ≥15.0 points, showing a lower risk of bias and higher quality of the studies. Both the assessment tools showed good results with regard to the quality of the selected studies.

The discrepancies between the evaluations within these two scales may be related to the objective for which each one was designed. While AXIS provides an evaluation of more general items,^24^ PEDro provides an assessment for more specific aspects in clinical trials,^25^ so the lowest PEDro score, especially in items related to blinding, can signal possible biases, like exaggerating or reporting fewer symptoms. These biases can induce different rates of, for example, co-intervention, friction, and placebo effect.^26,27^ Furthermore, our results suggest that future studies should exercise caution in the definition and methodological execution, so that the studies are in accordance with the guidelines of the designs to which they are proposed.

## DISCUSSION

There are significant increases in research aimed at identifying diagnostic and prognostic biomarkers of AD. The updated AD research structure proposed by the National Institute on Aging and Alzheimer’s Association working group defines AD as a biological construct, and the research focused on the diagnosis of AD in those who are alive using biomarkers that cover the presymptomatic and symptomatic stages of the disease. Biomarkers are grouped into those of Aβ deposition, pathological tau, and neurodegeneration. Although it is possible that Aβ plaques and neurofibrillary tau deposits are not the cause of AD pathogenesis, these abnormal protein deposits define AD as a unique neurodegenerative disease among various diseases that can lead to dementia.

The preclinical imaging methods used by diagnostic of amyloid accumulation and neurodegeneration (i.e., imaging PET) and biofluids (CSF and blood plasma) are very expensive and difficult to use in research. Therefore, the attention of researchers is directed to the search for predictors of the disease, which indirectly reflect the functional activity of the structures involved in the pathological process.

With regard to the prognostic value, the most relevant studies sought to identify biomarkers at early stages of AD pathogenesis, particularly the studies involving groups of healthy elderly individuals, characterized by the accumulation of cerebral amyloid plaques in the absence of clinical symptoms of mild cognitive impairment (MCI) or dementia. The hippocampus is a key brain region that processes EM and is a primary structure that is susceptible to the accumulation of amyloid plaques. However, some structures such as the entorhinal cortex and the cingulate gyrus are also relevant to the matter. Currently, some studies aimed at studying the relationship of minimal cognitive impairment, including the decline of EM, in different groups of subjects such as those with AD symptoms and/or MCI and the elderly with preservation of cognitive functions.^28,29,30^ The drawback is that the methods and procedures used to investigate EM vary and do not always accurately reflect EM. It is also important to mention that visual processing^31,32^ is one of the main biomarkers for cognitive decline.^33^

An episode can be classified as “what” happens “where,” with contextual information (temporal “when,” or circumstantial “which”) that fosters contextual and behavioral criteria.^34^ As follows, the hippocampal formation is necessary for learning and memory,^35,36,37^ particularly for spatial components.^38^ The trigger between pyramidal neurons in the CA1 and CA3 regions is clearly correlated with the location of an individual.^38,39^ The ablation of the hippocampal formation, in turn, impairs spatial navigation ability.

The tests such as CVLT and RCFT have considerable clinical predictive value and reliability and are widely used for the clinical assessment of EM.^16,17^ However, classic neuropsychological tests are limited to evaluating the recall of focal elements, such as lists of images or words, freely or with the use of cues.^18,19^ Thus, they are more associated with semantic or verbal processes than episodic processes, and they do not consider contextual elements.^19^ Most studies that were discussed in this review attempted to circumvent this hindrance by employing at least two types of classic neuropsychological tests to assess EM. However, the set of tests that are used by these studies may not provide an assessment that fully integrates “what,” “where,” and “when” components that are essential for predicting real memory performance in everyday life.^19,20^. Additionally, using a battery of tests can be tiring for respondents, especially for the elderly people. The Treasure Hunt task^20^ is proposed to investigate three components (i.e., what, where, and when) and be useful for further studies. This task was developed by Cheke^20^ and is proposed to assess the memory of object information (“what”), location information (“where”), and temporal order information (“when”) within the same testing paradigm. This is important because it integrates all three features into a classic what–where–when framework. This task can also identify the extent and pattern of EM deficits that might be present in several diseases. Although no normative data have been published for the Treasure Hunt task, its application can still be useful. It is difficult to identify whether there is a specific deficit of EM, and the Treasure Hunt task can be an important alternative or complement to the existing assessment tools.

The divergence between age and cognitive functioning may be grounded in the medial temporal lobe (MTL). The MTL, comprising interconnected structures of the hippocampus, dentate gyrus, peri- and entorhinal cortices, and parahippocampal gyrus, undergoes a prolonged period of postnatal development in humans, nonhuman primates, and rodents, with different maturation timelines. The MTL also impacts learning and memory functions differently in time.^40^ These areas have functions in general mnemonic processes, specifically EM in humans and rats. It has been suggested that the function of the hippocampus is to integrate details of events that have been experienced, including spatial locations, together to gather episodic memories.^41^

EM is a multisensory neurocognitive process of linking many elements. The unification of many elements in an episode occurs because of the long-term potentiation in the MTL and the activity of hippocampal neurons in the theta phase.^42,43^Studies have been demonstrated that theta cycles determined the process packaging of principal cell spiking into functional ensembles *via* the provision of discrete windows in which incoming streams of information from different systems are processed.^44,45^

In addition, there is ample evidence that the *N*-methyl-d-aspartate glutamatergic receptor has a fundamental function in inducing synaptic plasticity and memory formation for various tasks involving aversive conditioning,^47^ training of spatial memory,^48^ nonspatial, and nonaversive tasks^49^ in rodents^46^. These studies provided evidence that the deposition of neocortical and hippocampal Aβ in elderly people with normal cognitive aging is associated with functional changes in EM.^50^. In general, aging is associated with a reduced ability to modulate MTL activity, that is, with aging, the hippocampus shows a decrease in activation, and the entorhinal cortex decreases inhibition during an EM task. In addition, among elderly individuals, Aβ deposition was associated with a reduction in the entorhinal cortex regions associated with standard network functioning.^1,5,9^

It is strongly suggested that in elderly individuals with a high concentration of Aβ, the preclinical processes of AD have begun despite normal cognitive functioning, even in the absence of changes in clinical findings. It is important to mention that this can be detected over a short period of time through the use of neuropsychological measures.^51^Age and Aβ deposition contribute to a collapse of the network between the hippocampus and regions of the neocortex, suggesting the declines in EM.

Aging-related dementia usually begins gradually, with hippocampal atrophy manifesting several years after the onset of clinical symptoms. Aβ deposition is a part of the pathophysiology of AD, and therefore Aβ is more focused as a biomarker of AD. It is known that the role of Aβ in neurodegeneration culminates in a cascade of harmful events such as dementia and AD. However, this review noted that even in the presence of cognitive aging, Aβ can be detected in some individuals. The factors or issues that make individuals to be at risk of developing AD are still unclear.^51,52^

One key to improving our understanding of the relationship between normal aging and initial stages of AD is related to neuroplasticity and cognitive decline that results from a lack of a compensatory response to the accumulation of Aβ. The leading genetic risk factor for AD, namely, apolipoprotein ε4, is related to neural plasticity. The high levels of cognitive reserves are associated with the level of education and socioeconomic status of an individual and delay in the diagnosis of AD^53^. Individuals with greater cognitive reserves can maintain cognitive function in the face of the higher levels of cerebral Aβ deposition^54^. Weak correlations between the levels of Aβ deposition and cognitive function suggest that other mechanisms, such as functional compensation, influence cognitive ability. The views that cognitive function can be maintained during aging by compensatory cognitive processes and that this decline is seen when a person is no longer able to compensate for a decrease in the function of primary brain structures and circuits are well supported in the contemporary research.^55,56^

High-performing elderly adults are quite interesting in this regard. High-performing elderly individuals exhibit global preservation of the cerebral cortex, especially the anterior cingulate gyrus, and the volume of the hippocampus is higher than in people of normal age. The histological analyses of this group also revealed lower amyloid burden and lower neurofibrillary tangles compared with cognitively normal elderly controls. Thus, further studies of high-performing older adults are likely to provide additional insights in the role of amyloid deposition in the hippocampus during the aging process.^57^

Despite the strengths of this review, it has some limitations. First, this review included studies that involved age ranges, which created challenges in interpreting these results (e.g., age is directly associated with amyloid deposition). Second, several studies used tests that assessed only specific parts of EM (e.g., delayed recall), thus raising the need to use extensive neuropsychological assessments or tasks, such as the Treasure Hunt task, that encompass all three main aspects of EM. Third, the analysis of various brain regions raises the need to define possible neural networks that are responsible for EM processing.

Finally, we noted the need for further research in this area. It is necessary to investigate this concept in order to understand the prodromal symptoms of AD and the emergence of new practices and techniques that can identify and map Aβ deposition during healthy aging.
